# One-year post lockdown trajectories of mental health and impact of COVID-19 lockdown-related factors

**DOI:** 10.3389/fpubh.2025.1457895

**Published:** 2025-03-12

**Authors:** Mathilde Mongeau, Samantha Huo Yung Kai, Vanina Bongard, Nicola Coley, Emilie Bérard, Jean Ferrières

**Affiliations:** ^1^Department of Epidemiology and Public Health, Toulouse University Hospital, Toulouse, France; ^2^Artherosclerosis Risk and Treatment Evaluation Towards Risk Reduction Epidemiology (ARTERRE) Axe, Centre for Epidemiology and Research in Population Health (CERPOP), INSERM-University of Toulouse UPS, Toulouse, France; ^3^Department of Cardiology, Toulouse University Hospital (CHU), Toulouse, France; ^4^Aging Research Team, Centre for Epidemiology and Research in Population Health (CERPOP), INSERM-University of Toulouse UPS, Toulouse, France; ^5^Institut Hospitalo-Universitaire (IHU) HealthAge, Toulouse, France

**Keywords:** COVID-19, lockdown, mental health, general population, 1-year follow-up, France

## Abstract

**Introduction:**

Concerns about the impact of restrictive measures on people’s wellbeing, especially mental health, were raised by the COVID-19 pandemic and related lockdown measures.

**Methods:**

In this study, we examined longitudinal trajectories of mental health during the initial French lockdown period and up to one-year post-lockdown, among a representative sample of French adults aged over 50. We also assessed the impact of COVID-19 lockdown-related factors on mental health. A cohort of 534 individuals was enrolled during the first French lockdown in March 2020, and four telephone interviews were conducted during the lockdown, and at 1-, 6- and 12-months post-lockdown. Mental health was assessed using validated scores of anxiety and depression (GAD-7 and PHQ-9, respectively). Participants undergoing treatment for anxiety or depression at inclusion were excluded.

**Results:**

Our analysis revealed a significant decrease in the proportion of individuals experiencing poor mental health (elevated GAD-7 or PHQ-9 > 4) from lockdown period to 1 month and 6 months post-lockdown. However, this improvement stopped at 12 months post-lockdown, likely reflecting the reinstatement of strict measures in 2021. We used logistic regression to identify factors independently associated with early and long-lasting deterioration in mental health (elevated GAD-7 or PHQ-9 > 4 at first or second interview that persisted over at least two interviews). History of anxiety, poor perceived global health, female gender, working during lockdown, not being in a relationship, and having a relative suspected of being COVID-positive were significantly associated with deterioration in mental health.

**Discussion:**

Our study highlights factors associated with a mental health impact during and following a lockdown in a representative sample of people, aged over 50 years old, thus at increased risk of severe COVID-19 and more likely to be subject to lockdown measures. These factors could be targeted in public health actions in future pandemics.

## 1 Introduction

The emergence of the COVID-19 pandemic in late 2019 led to a global public health crisis with unprecedented healthcare systems overload. In response and due to the rapid spread of the virus, the French government imposed a strict lockdown from March 17, 2020, to May 11, 2020. In France, daily outdoor activities were restricted to essential needs such as grocery shopping or other proven necessities. After the initial lockdown, the French government implemented several other measures to contain viral transmission, including two additional lockdowns in November 2020 (from 30 October 2020 to 28 November 2020) and April 2021 (from 2 April 2021 to 2 May 2021), and two national curfews in December 2020 (from 15 December 2020 to 2 April 2021) and May 2021 (from 3 May 2021 to 20 June 2021). While these measures were crucial in limiting viral transmission and protecting the most vulnerable people from infection, a growing body of scientific evidence shows a significant negative impact on mental health.

Longitudinal studies has shown heterogeneous mental health reactions through lockdown and in the following time period. Psychological impact was not limited to lockdown period and those symptoms lasted, even after the end of restrictions. While the majority of people showed resilience, more vulnerable people showed a deterioration of mental health during or shortly after lockdown period (1–4).

Many symptoms may be associated with a decline in mental health including depression, anxiety, sleep disturbance, stress, and irritability. For instance, the quality of relationships appears to be a critical factor in maintaining good mental health (5, 6). In contrast, people with financial difficulties (7, 8), confined to an apartment (or a house without a garden or balcony) (9), and alone (7, 10) were more likely to develop depressive symptoms during lockdown. Existing research has shown that those most affected by the pandemic, and in particular by the lockdown, were the older adults (11–13), people with a history of mental disorder (1, 7, 14–19), children (11), women (1–3, 11, 12, 14, 15, 19–27), precarious workers, students or non-working people (2, 15, 16, 22, 28), and people living in small (11, 29) or poor housing (24, 30).

Several studies have examined the impact of people’s characteristics on the mental health during lockdown or shortly after lockdown (2, 3, 6, 7, 10, 11, 13–17, 20, 21, 26, 28, 31). Nevertheless, to our knowledge, no study has assessed the impact of COVID-19 lockdown with a 12-month follow-up.

This study aimed to examine the impact of the COVID-19 pandemic and of the national lockdown imposed in France on mental health in a representative sample of the middle-aged and older French general population. Specifically, we describe mental health trajectories with a 1-year post lockdown follow-up and examine individual characteristics associated with a deterioration in mental health from pre-lockdown.

## 2 Materials and methods

### 2.1 Study population

The results presented in this paper are secondary analyses from the original PSYCOV-CV dataset which has been already described previously (32, 33). A sample of 534 individuals was enrolled in the PSYCOV-CV cohort (NCT04397835) during the first French lockdown (from 17 April 2020 to 10 May 2020). Participants were 50–89 years old and had previously participated in the south-western France (Toulouse area) MONALISA cross-sectional population-based study on the prevalence of cardiovascular risk factors between 2005 and 2008 (34, 35). In the MONALISA study, participants were men and women, 35–74 years old and recruited using polling lists (available at each town hall in the study area) to obtain a stratified random sample. Stratification was applied by town size (rural versus urban), age, and gender to obtain 200 participants in each gender and 10-year age group (35–44, 45–54, 55–64, 65–74 years). No financial incentive for participation was offered. During the initial French lockdown, participants of the MONALISA study were contacted again to take part in the PSYCOV-CV cohort study. The participation rate in PSYCOV-CV was 69%. All subjects gave informed consent prior to participation in the MONALISA and PSYCOV-CV studies. The MONALISA and PSYCOV-CV studies were conducted in accordance with the Declaration of Helsinki, and the PSYCOV-CV protocol was approved by the Ethics Committee in April 2020 [“Comité de Protection des Personnes (CPP),” Ile de France V, France: protocol code 20.04.03.46101, date of approval April 3, 2020].

### 2.2 Telephone interviews

Trained researchers conducted telephone interviews during the first French lockdown (from 17 April 2020 to 10 May 2020) and at 1-, 6- and 12-months post-lockdown. The questionnaires were extensive and included both prospective data related to the lockdown period and retrospective data about the last 2–4 weeks before lockdown. We recorded data on several factors including socio-economic status, personal and familial medical history, cardiovascular risk factors, lifestyle habits, drug use, and specific lockdown-related factors such as living in a rural or urban environment, the number of people living with the participant during lockdown, having an exterior in the lockdown living space, and their feelings about the lockdown. We assessed perceived health status (good / good but not perfect / bad), difficulties in grocery shopping during lockdown, being infected with COVID-19 (or having a relative infected), perceived level of risk of COVID-19 infection (on a scale from 0 to 10), feelings of isolation, and being in a relationship. The participants’ educational level and professional activity were also assessed (in particular, having a professional activity during lockdown), together with smoking status (and the daily consumption of cigarettes and cigarillos) and alcohol consumption (using a 7-day recall method for a typical week). The assessment of physical activity outside work (considering the travel to and from work, sport or any other physical effort during leisure time, such as gardening or dancing…) used 4 levels “No weekly physical activity,” “Only light physical activity almost every week,” “Intense physical activity for at least 20 min once or twice a week (intense physical activity causes shortness of breath, rapid heartbeat and sweating),” “Intense physical activity for at least 20 min three or more times a week.” Duration (in minutes) was also assessed for a typical workout session. Sedentary lifestyle was assessed in hours/day for screen time on TV, computer, or smartphone. The time spent on average during a usual week inside the house doing moderately intense to very intense household chores (for example: vacuuming, cleaning floors or anything else requiring similar effort) was assessed. The participants self-reported their height and weight. Body mass index (BMI) was calculated by dividing the weight in kilograms by height in meters squared. Symptoms of anxiety and depression were evaluated using validated scales for the general population (36, 37): the Generalized Anxiety Disorder-7 (GAD-7) scale (No anxiety: 0–4 points; Mild anxiety:5–9 points; Moderate anxiety: 10–14 points; Severe anxiety: 15–21 points) and the Patient Health Questionnaire-9 (PHQ-9; No depression: 0–4 points; Mild depression:5–9 points; Moderate depression: 10–14 points; Severe depression: 15–21 points).

More details on the construction of this cohort and the interview framework can be found in the original papers on the PSYCOV-CV cohort published in 2021 and 2022 (32, 33).

### 2.3 Outcomes

Poor mental health at each interview was defined as an anxiety score (GAD-7) greater than 4 (indicating at least mild anxiety) or a depression score (PHQ-9) greater than 4 (indicating at least mild depression). To examine longitudinal worsening of mental health, we defined early and long-lasting worsening of mental health if poor mental health was observed at the first (i.e., at baseline) or second interview and last for at least two consecutive interviews. In order to consider only incident cases that occurred during the 12-month post-lockdown period, participants with current anxiety or depression at enrollment were excluded. Therefore, subjects who had received physician-prescribed medication for depression or anxiety in the 2 weeks prior to lockdown were excluded. Consequently, 39 participants were excluded which led to a total study population of 495 subjects for these analyses.

### 2.4 Statistical analysis

Statistical analyses were performed using STATA statistical software, version 18.0 (STATA Corporation, College Station, TX, United States). We first describe the main characteristics of the participants. In order to assess if there was a significant difference between proportions of people with a poor mental health at each interview post-lockdown compared to during lockdown, we ran McNemar chi2 tests, allowing comparison of paired percentages. The association between each variable of interest and longitudinal mental health deterioration (i.e., early and long-lasting mental health deterioration) was first assessed in bivariate analysis. For the binary variable of deterioration in mental health, i.e., deterioration of the GAD-7 score (assessing anxiety), or the PHQ-9 score (assessing depression), we had to manage missing data. Indeed, not all subjects had completed all 4 mental health assessments (99 subjects had at least one of the 4 planned assessments missing, or 20% of participants). To avoid losing these subjects in the analysis, we used a last observation carried forward method to handle missing data. Therefore, we assumed that a subject with poor mental health (in terms of anxiety or depression) at one interview had a high probability of still having this worsening at the following interview. Then, multivariate logistic regression was used to assess which lockdown-related factors were independently and significantly associated with early and long-lasting worsened mental health. All data were tested with a single model and all predictors. Variables initially included in the multivariate analyses were associated with the mental health endpoint in bivariate analyses with a *p*-value <0.20 (See results of bivariate tests in Supplementary Table 4). When the linearity hypothesis was not verified, continuous variables were transformed into ordinal variables using quartile distributions. In order to detect multicollinearity, we used Variance Inflation Factors (VIF) with a cutoff of 5. A stepwise selection procedure was then used to sequentially remove variables with a *p*-value >0.05 from the multivariate model, starting with the variable with the highest p-value. We stopped the procedure when all the variables were significatively associated with the dependent variable. At each step of the procedure, we assessed the model’s fit using the likelihood ratio test to compare the fit of the previous model against the nested model. If the removal of a variable did not significantly decrease the model’s fit, the variable was removed from the model. Interactions between independent covariates were tested in the final model. None of the interactions were found to be significant. Finally, to assess the goodness of fit of the multivariate regression, we used the pseudo R2 value which quantifies the proportion of variability in the dependent variable explained by the independent variables in our models. All reported *p*-values were two-sided, and the significance threshold was <0.05.

## 3 Results

### 3.1 Characteristics of participants

Of the 495 participants included, 52% were female and the mean age was 66 years (±10). Participants’ baseline characteristics are described in Table 1. A quarter of our population declared a history of anxiety. Most participants were in a relationship during lockdown (71.9%) and had a level of education exceeding high school (87.9%). More than half of our population declared consuming alcohol at least once a week during lockdown (63.3%), while 64% considered themselves to be in good health before lockdown.

**Table 1:** Characteristics of the participants before and during the COVID-19 lockdown (17 March 2020 to 10 May 2020, France).

Characteristics	Total *N* = 495
Age (in years) during lockdown, mean (SD)	66.49 (10.41)
Female gender, n (%)	255 (51.5)
BMI before lockdown, mean (SD)	25.6 (4.5)
History of anxiety^a^, n (%)	128 (25.9)
History of depression^b^, n (%)	26 (5.3)
Being in a relationship during lockdown, n (%)	356 (71.9)
Obesity before lockdown, n (%)	48 (9.7)
Educational level: at least high-school completion, n (%)	435 (87.9)
Smokers before lockdown, n (%)	59 (11.9)
Number of cigarettes per day before lockdown^c^, mean (SD)	0.84 (3.23)
Consuming alcohol (at least once a week) during lockdown, n (%)	307 (63.3)
Good health status before lockdown^d^, n (%)	317 (64.0)
Having an outdoor space (garden or balcony) during lockdown, n (%)	483 (97.8)
Feeling of isolation during lockdown^e^, n (%)	90 (18.2)
Professionally active during lockdown, n (%)	343 (69.3)
Being physically active before lockdown^f^, n (%)	276 (55.8)
Duration (in minutes) of a typical workout session before lockdown, mean (SD)	87.03 (61.01)
Increased sedentary lifestyle during lockdown^g^, n (%)	266 (53.7)
Variation of time used to do household chores since lockdown^h^	
*No change*	279 (57.8)
*Augmentation*	143 (29.6)
*Reduction*	61 (12.6)
Difficulties to do groceries during lockdown^i^, n (%)	31 (6.4)
Being COVID-positive (or suspected) during lockdown^j^, n (%)	23 (4.7)
Having a COVID-positive (or suspected) relative during lockdown^k^, n (%)	60 (12.4)
Estimated level of risk of contamination (on a scale from 1 to 10), mean (SD)	3.69 (2.26)

SD, Standard Deviation.

a History of anxiety was assessed by the question “Did you have any anxiety issue?”.

b History of depression was assessed by the question “Did you have any depression issue?”.

c The number of cigarettes was 0 for non-smokers and included cigarillos.

d Good health condition is a binary variable created from the declaration answer at the following question “Do you consider yourself in good health since lockdown?” and for which answers were “yes,” “yes but not perfectly,” “no.” Both answers with “yes” were grouped together.

e Feeling of isolation was assessed by the question “Do you currently feel isolated socially?” (answers from never to constantly).

f Being physically active before lockdown is a binary variable created from the declaration answer at the following question “Before lockdown, which of the following four conditions best descried your physical activity outside work? Consider the travel to and from work, sport or any other physical effort during your leisure time, such as gardening or dancing…” and for which answers were “No weekly physical activity,” “Only light physical activity almost every week,” “Intense physical activity for at least 20 min once or twice a week (intense physical activity causes shortness of breath, rapid heartbeat and sweating),” “Intense physical activity for at least 20 min three or more times a week.” Both answers with “intense physical activity” were considered as being physically active while “no physical activity” and “light physical activity” were considered as being physically inactive.

g Increased sedentary time was considered if the time spent per day in front of the TV or a computer screen or a smartphone has increased between before and during lockdown.

h Represents the variation of time spent on average during a usual week inside the house doing moderately intense to very intense household chores during lockdown compared to before lockdown (for example: vacuuming, cleaning floors or anything else requiring similar effort).

i Difficulties to do groceries during lockdown refers to the answer to the following question “Since lockdown, have you had any difficulty doing your groceries?”.

j Being positive to COVID-19 (or suspected) is the answer to the following question “Has a doctor ever told you that you have or suspect COVID-19?,” asked during lockdown.

k Relative positive to COVID-19 (or suspected) is the answer to the following question “Has a doctor ever told a relative that they have or suspect COVID-19?,” asked during lockdown.

### 3.2 Trajectories of poor mental health during the 1-year post-lockdown follow-up

**Figure 1:**
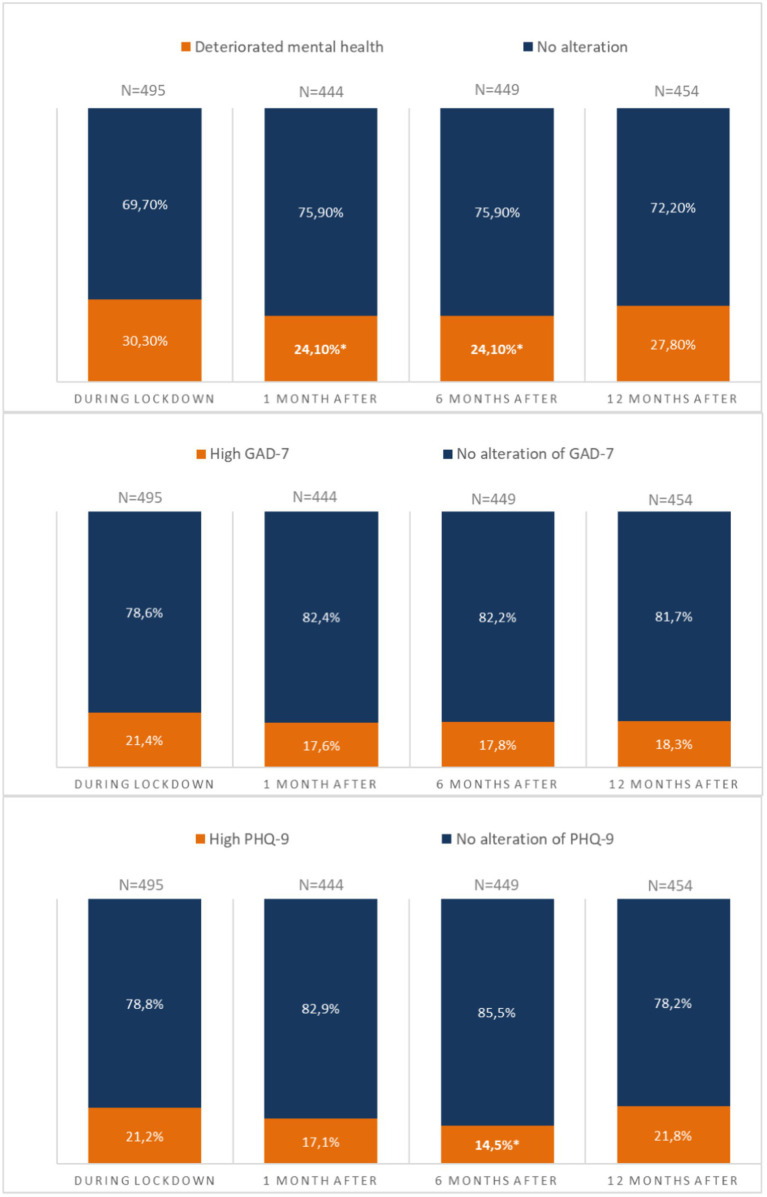
Trajectories of poor mental health [GAD-7 anxiety score > 4 or PHQ-9 depression score > 4] during the 1-year post-lockdown follow-up. *Significant differences compared to during lockdown; GAD-7, Generalized Anxiety Disorder-7 (No anxiety: 0–4 points; Mild anxiety:5–9 points; Moderate anxiety: 10–14 points; Severe anxiety: 15–21 points); PHQ-9, Patient Health Questionnaire-9 (No depression: 0–4 points; Mild depression:5–9 points; Moderate depression: 10–14 points; Severe depression: 15–21 points).

In our sample, approximately one third of subjects experienced poor mental health during the initial lockdown. Figure 1 shows the trajectories of poor mental health [GAD-7 anxiety score > 4 or PHQ-9 depression score > 4] during the 1-year post-lockdown follow-up. The proportion of subjects with poor mental health was significantly less 1- and 6-months post-lockdown compared to during lockdown. While global mental health seems to improve 1 month and 6 months after the initial lockdown, the enhancement stopped at 12 months post-lockdown.

Table 2 shows the distribution of participants by trajectory of poor mental health during the 1-year follow-up period.

**Table 2:** Description of trajectories of poor mental health [GAD-7 anxiety score > 4 or PHQ-9 depression score > 4].

	Poor mental health during lockdown				Total		*p*-value
	Yes		No				
	n	%	n	%	n	%	
Poor mental health at 1-month follow-up							
Yes	69	52.7	38	12.1	107	24.1	0.016
No	62	47.3	275	87.9	337	75.9	
Poor mental health at 6-month follow-up							
Yes	69	50.4	39	12.5	108	24.1	0.005
No	68	49.6	273	87.5	341	75.9	
Poor mental health at 12-month follow-up							
Yes	77	54.6	49	15.7	126	27.8	0.158
No	64	45.4	264	84.3	328	72.2	

GAD-7, Generalized Anxiety Disorder-7 (No anxiety: 0–4 points; Mild anxiety:5–9 points; Moderate anxiety: 10–14 points; Severe anxiety: 15–21 points); PHQ-9, Patient Health Questionnaire-9 (No depression: 0–4 points; Mild depression:5–9 points; Moderate depression: 10–14 points; Severe depression: 15–21 points).

Of the 126 participants with deteriorated mental health 12 months after lockdown (27.8% of the sample), 52% (*N* = 65) were already experiencing deteriorated mental health 6 months after lockdown, 50% (*N* = 63) were already experiencing deteriorated mental health 1 month after lockdown and 61.1% (*N* = 77) were already experiencing deteriorated mental health during confinement. In the end, 38 participants (7.7% of the sample) experienced deteriorated mental health from the lockdown through all the 12-month follow-up period.

### 3.3 Determinants of worsening mental health during the 1-year post-lockdown follow-up

To examine longitudinal worsening of mental health, we defined early and long-lasting worsening of mental health if poor mental health was observed at the first (i.e., at baseline) or second interview and last for at least two consecutive interviews. An overview of results of bivariate tests is available in Supplementary materials. Only variables with a *p*-value of less than 0.2 in the associated bivariate tests were included in the initial multivariate model.

Table 3 shows factors independently and significantly associated with early and long-lasting worsening of mental health. Notably, female gender, and a history of anxiety were associated with more frequently deterioration of mental health [adjusted OR = 2.38 (1.42–3.97), *p* = 0.001, and aOR = 4.40 (2.61–7.41), *p* < 0.001, respectively]. Furthermore, people declaring themselves in a good health condition before lockdown and in a relationship during lockdown were less likely to worsen their mental health during the follow-up period [aOR = 0.42 (0.25–0.71), *p* = 0.001, and aOR = 0.40 (0.23–0.69), *p* = 0.001, respectively]. In the contrary, participants with a professional activity during lockdown and having difficulties to do groceries during lockdown were at higher risk to worsen their mental health post-lockdown [aOR = 2.37 (1.39–4.05), *p* = 0.002, and aOR = 2.90 (1.89–7.05), *p* = 0.019]. Similarly, people with a relative positive to COVID-19 (or suspected) and having less time to do household chores since lockdown were more likely to worsen their mental health during the period [aOR = 2.11 (1.05–4.20), *p* = 0.035, and aOR = 2.05 (1.01–4.17), *p* = 0.048, respectively].

**Table 3:** Factors independently and significantly associated with early and long-lasting worsening of mental health [GAD-7 anxiety score > 4 or PHQ-9 depression score > 4] (*N* = 110/491).

	Odds-ratio (OR)	95% confidence interval	*p*-value
History of anxiety^a^	4.40	2.61–7.41	<0.001
Good health condition before lockdown^b^	0.42	0.25–0.71	0.001
Female gender	2.38	1.42–3.97	0.001
Professional activity during lockdown^c^	2.37	1.39–4.05	0.002
Difficulties to do groceries during lockdown^d^	2.90	1.89–7.05	0.019
Relative positive to COVID-19 (or suspected)^e^	2.11	1.05–4.20	0.035
Being in a relationship during lockdown	0.40	0.23–0.69	0.001
Variation of time used to do household chores since lockdown^f^			
No variation	1	–	–
Increase	0.72	0.40–1.30	0.272
Decrease	2.05	1.01–4.17	0.048
Pseudo-R2 = 0.2027			

a History of anxiety was assessed by the question “Did you have any anxiety issue?”.

b Good health condition is a binary variable created from the declaration answer at the following question “Do you consider yourself in good health since lockdown?” and for which answers were “yes,” “yes but not perfectly,” “no.” Both answers with “yes” were grouped together.

c Professional activity during lockdown includes people with a job (out of home) with and without in-person contact with the public during lockdown, as well as teleworking.

d Difficulties to do groceries during lockdown refers to the answer to the following question “Since lockdown, have you had any difficulty doing your groceries?”.

e Relative positive to COVID-19 (or suspected) is the answer to the following question “Has a doctor ever told a relative that they have or suspect COVID-19?,” asked during lockdown.

f Represents the variation of time spent on average during a usual week inside the house doing moderately intense to very intense household chores during lockdown compared to before lockdown (for example: vacuuming, cleaning floors or anything else requiring similar effort).

All non-collinear significant factors (at the 20% threshold in bivariate analysis) were included in the initial model. The final model was obtained using a descending stepwise method.

## 4 Discussion

We found that the proportion of subjects with poor mental health was significantly lower 1- and 6-months post-lockdown compared to during the lockdown. While global mental health appeared to improve 1 month and 6 months after the initial lockdown, the improvement stopped at 12 months post-lockdown. This dynamic was also found in a British study assessing the impact of lockdown on referrals to secondary care mental health clinical services (38). After an initial drop in referrals after lockdown, they observed a post-lockdown acceleration in urgent and emergency mental health referrals. European studies assessing the evolution of mental health during and after the lockdown showed a gradual improvement in depression and anxiety after the end of lockdown measures, probably because individuals adapted to circumstances (39–42). In France, 1 year after the first lockdown, there was a reinforcement of health measures with the maintenance of curfews, closure of socializing places such as bars, restaurants, and cultural places during the winter of 2020–2021. This period was also followed by a third lockdown in spring 2021, characterized by severe restrictions such as limited travel within a 10 km radius and school closures. This tightening of nationally imposed rules appears to be consistent with the mental health of the subjects included in the study, similar to that evaluated during the initial lockdown (March 2020).

As anticipated, we found that female gender and history of anxiety were associated with a higher probability of deterioration of mental health during and after lockdown. Our results are consistent with previous findings in the literature showing that females and people with history of anxiety are more likely to worsen mental health during lockdown periods (1–3, 11, 12, 14, 15, 19–27). Thus, it seems crucial to increase accessibility to mental health services to mitigate the effects of lockdown, especially on patients with a history of mental health disorders. Indeed, our final model has a pseudo-R2 of 0.2027. This result indicates that the variables included in the model contribute 20.27% to the understanding of the phenomenon studied. As expected, the history of anxiety is a key factor in the regression and its withdrawal causes a drop in the pseudo-R2 to 0.1423. Moreover, we found that participants who were professionally active during lockdown, had difficulties to do groceries during lockdown, had a relative who tested positive to COVID-19 (or suspected), and who had less time to do household chores since the start of lockdown were more likely to worsen their mental health early and durably after the beginning of the lockdown. Those who maintained professional activities throughout lockdown (including working with or without in-person contact and teleworking) may have found it difficult to prioritize personal wellbeing and leisure activities, given that their work obligations persisted. Indeed, previous research showed a significant correlation between participation in meaningful activities and reduced levels of psychological distress (24, 29). In addition, having a job during lockdown was found to be a predictor of mental health symptoms in an Italian study (31). People whose work involves contact with the public were most exposed to COVID-19 (sometimes isolated from their families to avoid contamination). This included medical and paramedical professionals, as well as supermarket workers, whose working conditions were extremely difficult in this context. Nevertheless, we have to notice that we identified that teleworkers were more likely to worsen their mental health among all workers. This may be linked to a drop in social relations. More broadly, an Italian study that followed bank employees for a year post-lockdown showed that massive adjustments to a work or a family routine were a significant source of stress and possibly of people’s wellbeing (43). Additionally, some stores experienced shortages of certain foods during the pandemic. This may have caused stress and anxiety among people who feared they would not be able to find food to feed their families. Another source of stress may have been having to wait in line for long periods of time to access grocery stores. Regarding people with a COVID-19 diagnosis for themselves or their relatives, our results are consistent with another French study showing that these individuals are at greater risk of depression (26). On the contrary, participants in a relationship during lockdown and declaring themselves in good health since lockdown were less likely to worsen their mental health early and durably after the beginning of the lockdown. Perceived global health has been found as a key protective factor to maintain a good quality of mental well-being during and after the lockdown, as it was shown in various studies (1, 17, 29). Our results are also consistent with a British study assessing long-term psychological distress after lockdown: the highest was among younger people, women, people living without a partner, those who had no work or lost income, and those with previous health conditions or COVID-19 symptoms (2). Finally, people with less time to do household chores were more likely to worsen their mental health. For this variable, we cannot determine which phenomenon appeared first: the mental health deterioration or the decline of time used to do household chores. Indeed, doing less chores could be a precursor of a certain mental health decline.

Perceived risk of being infected by COVID-19 was not included in our final model, while it was found to be a major predictor of depressive symptoms in other studies (21, 44). Similarly, access to a garden or a balcony was found as a protective factor to a deterioration of mental health in other studies, in Thailand for instance (9). While this factor was included in our initial model, it did not remain in our final model, probably because of lack of power with a high percentage of our population having access to an exterior during the lockdown (97.8%).

Our study has some limitations that need to be considered. First, the results are based on self-reported data collected through telephone interviews. We had no other choice but to proceed to telephone interviews, instead of face-to-face interviews due to lockdown measures but telephone interviews may have encouraged participants to show a more positive self-image than reality. Among all data collected during these interviews, some variables relied on retrospective self-reports (e.g., recalling food consumption over the past weeks). This method could introduce recall bias, as participants might not accurately remember or report past behaviors. Secondly, we probably limited the population included in the endpoint “early and long-lasting worsening of mental health” by using a strict definition of mental health (we proxied mental health by anxiety symptoms using the GAD-7 questionnaire and depression symptoms using the PHQ-9 questionnaire) and ignoring others mental health symptoms such as sleep disturbance, feelings of guilt, mood changes, delusions, or anger. Mental health is multifaceted and we could have increased the number of individuals concerned by psychological distress by considering mental well-being as a whole instead of focusing on purely medical mental health (45). Additionally, the study may not be fully representative of the overall French general population since it was conducted in only one area of France (south-western France) and participants were 50–89 years old. It could be an important limitation about our results as another study showed that the mental health of the older adults was less impacted by lockdown periods than others group ages (46) and a Korean study showed that older people were less likely to experience mental health issues because of a better understanding of public health measures (47). The age group of our participants may also have influenced the fact that we did not include in our final model any variable about physical activity, while many studies showed a positive impact of exercise on mental health (23, 48). Finally, considering people with early and long-lasting mental health deterioration is also a limitation since people with late or short mental health issues could also benefit from public health interventions.

Nevertheless, our study has many strengths. First, the PSYCOV-CV data represents the first French observational prospective data on the impact of the COVID-19 lockdown on mental health, with a 12-months follow-up period. Furthermore, our study is representative of the middle-aged population living in the southwestern French area. This region of France was less affected by the pandemic (compared to the Northeast, for example), but we still saw a significant deterioration in mental health. Moreover, telephone interviews allow participants to feel more comfortable talking about their mental health with a stranger than in a face-to-face interview. This suggests that the answers given are more likely to reflect reality. Finally, the sample size of 495 individuals yielded highly clinically and statistically significant results. Given the impossibility of randomized controls, in a study on the impact of a national politically imposed lockdown, the results were adjusted to account for the main confounders.

Critically, people who are unable to buffer long-term exposure to stress are vulnerable to a range of negative health outcomes, including worsening mental health. Even worse, chronic psychological distress has been linked to lower levels of immunity and, as a result, increased susceptibility to the common cold, influenza, infectious diseases, and upper respiratory illnesses (49). It is crucial as measures to mitigate the COVID-19 pandemic could increase risk factors for coronavirus infection if these measures induce stress in the general population and thus have a large counterproductive effect. Consequently, the impact of psychological distress could at least partly offset beneficial health consequences of government measures (50). Indeed, it is imperative to be able to detect vulnerable people to adapt public health measures during crisis period, such as the COVID-19 associated lockdown, as a degraded mental health can led to undesirable behaviors, particularly in terms of sleep habits, physical activity, and quality of diet (51).

## 5 Conclusion

The proportion of subjects with poor mental health was significantly lower 1- and 6-months post-lockdown compared to during lockdown, but this improvement stopped at 12 months post-lockdown, likely reflecting the reinstatement of strict measures in 2021. We identified factors independently associated with early and long-lasting deterioration in mental health with 1-year follow-up, including a history of anxiety, poor perceived global health, female gender, working during lockdown, not being in a relationship, and having a relative suspected of being COVID-positive. We believe that our results increase the understanding of the mental health impact of the lockdown, in a representative sample of people over 50 who are at increased risk of severe COVID-19, and thus could be subject to additional lockdown periods. These factors could be targeted in future public health actions during any new pandemics.

## Data Availability

The raw data supporting the conclusions of this article will be made available by the authors, without undue reservation.
