# Supplementary figures and images for: One-year post lockdown trajectories of mental health and impact of COVID-19 lockdown-related factors
Source: Front Public Health. 2025 Mar 12;13:1457895. doi: 10.3389/fpubh.2025.1457895 (PMC11936785; doi:10.3389/fpubh.2025.1457895)

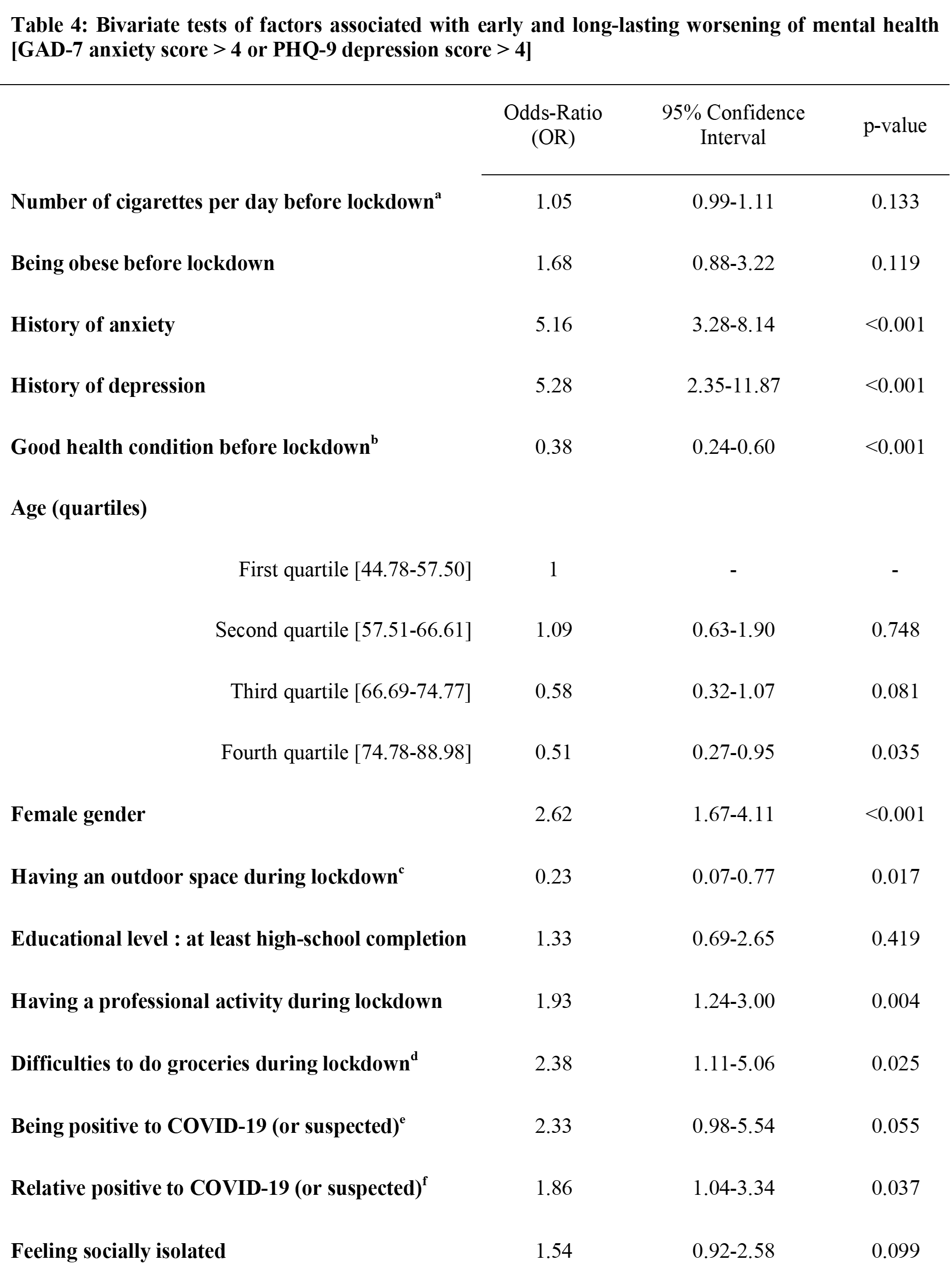

Supplement: Supplementary file 1 [file Image_1.PNG]

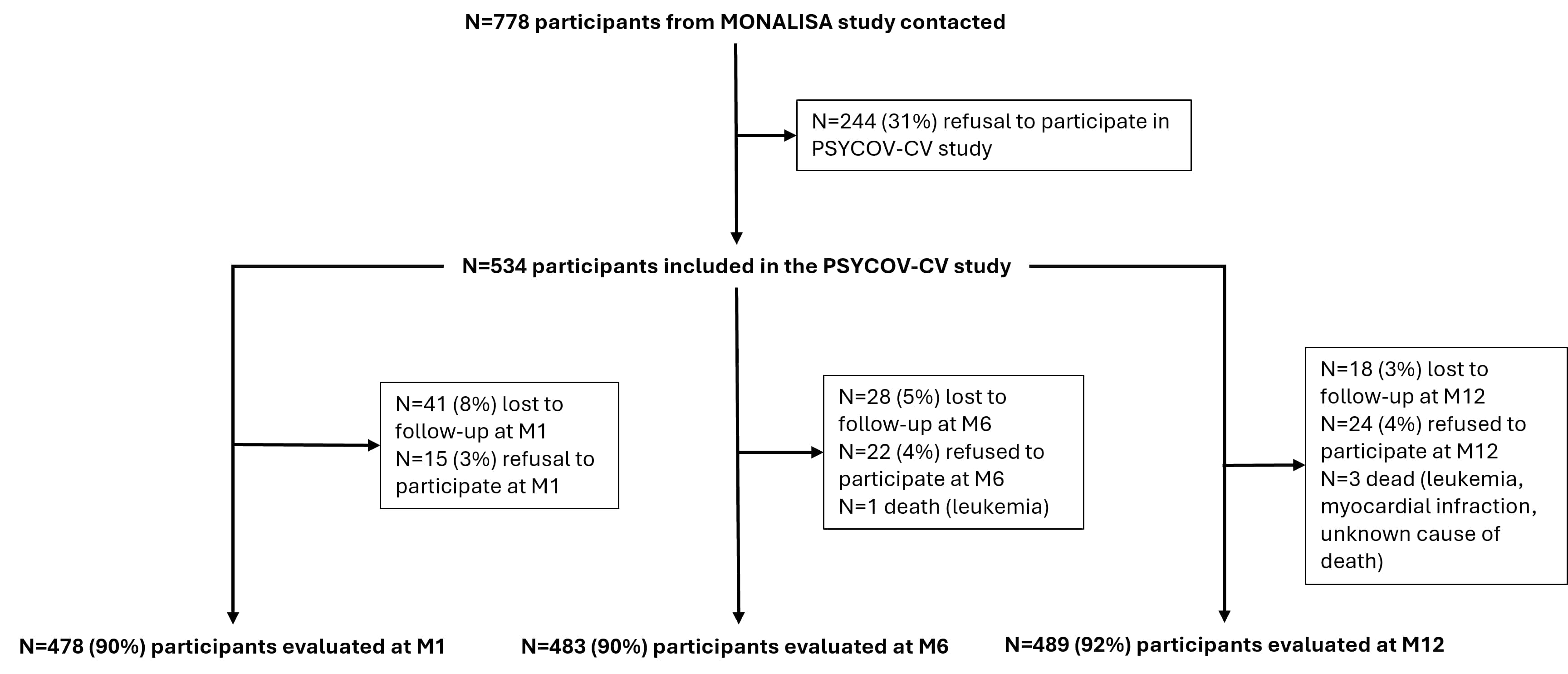

Supplement: Supplementary file 2 [file Image_2.JPEG]
